# Study of the influence of 2.5% Mg^2+^ insertion in the B-site of La_0.8_Ca_0.1_Pb_0.1_FeO_3_ on its structural, electrical and dielectric properties†

**DOI:** 10.1039/d1ra04041c

**Published:** 2021-10-07

**Authors:** H. Issaoui, A. Benali, F. Issaoui, E. Dhahri, B. F. O. Costa, M. P. F. Graca, M. A. Valente, Mohamed Lamjed Bouazizi

**Affiliations:** University of Coimbra, CFisUC, Physics Department P-3004-516 Coimbra Portugal; Laboratoire de Physique Appliquée, Faculté des Sciences, Université de Sfax B.P. 1171 3000 Sfax Tunisia; I3N and Physics Department, University of Aveiro 3810-193 Aveiro Portugal; Research Unit Valorization and Optimization of Resource Exploitation, Faculty of Science and Technology of Sidi Bouzid, University of Kairouan Sidi Bouzid 9100 Tunisia; College of Engineering, Prince Sattam Bin Abdulaziz University 655 Al Kharj 11942 Saudi Arabia

## Abstract

This work involves the synthesis and study of physical properties of the La_0.8_Ca_0.1_Pb_0.1_Fe_0.975_Mg_0.025_O_3_ compound, which has been characterized by various experimental techniques, such as X-ray diffraction, SEM and complex impedance spectroscopy. The structural study showed that the La_0.8_Ca_0.1_Pb_0.1_Fe_0.975_Mg_0.025_O_3_ compound crystallized in the orthorhombic structure with the *Pnma* space group. The particle size and the surface morphology of this compound have been analysed using SEM. The particle size was found to be around 120 nm and we confirmed that one particle contains more than one crystallite. Importantly, the studied compound presented a giant dielectric permittivity (*ε*′ of around 9 × 10^4^ at high temperature and low frequencies). An equivalent electric circuit has been deduced from the Nyquist plots of the complex impedance parts (*Z*′′ *vs. Z*′) to correctly describe the electrical behavior of the La_0.8_Ca_0.1_Pb_0.1_Fe_0.975_Mg_0.025_O_3_ compound. The chosen circuit consists of two cells mounted in series corresponding to the grain and grain boundary contributions. The electrode contribution has been detected from the frequency dependence of the imaginary part of modulus where the activation energy of each constitution has been calculated. The relaxation process and the electrical conductivity are attributed to the same type of charge carriers characterized by similar values of the activation energy determined from loss factor tangent (tg(*δ*)), the imaginary part of the permittivity and the modulus spectrum.

## Abstract 2

This work involves the synthesis and study of physical properties of the La_0.8_Ca_0.1_Pb_0.1_Fe_0.975_Mg_0.025_O_3_ compound, which has been characterized by various experimental techniques, such as X-ray diffraction, SEM and complex impedance spectroscopy.

## Graphical abstract

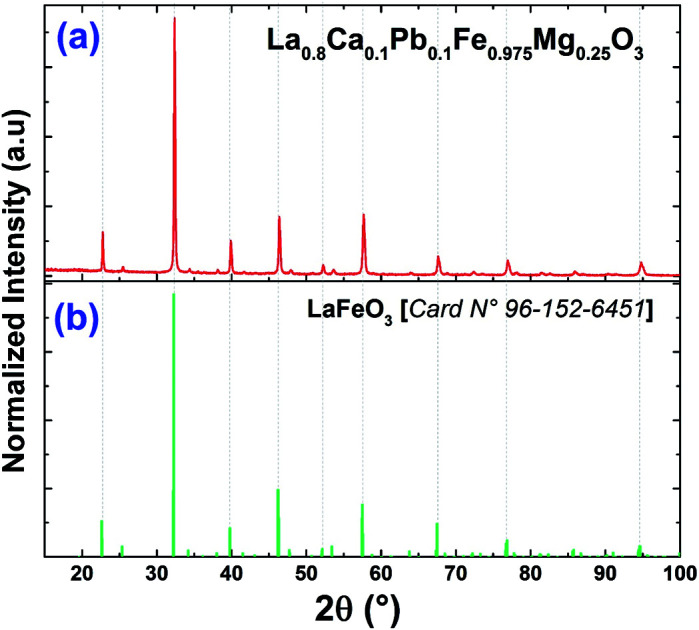

## 1 Introduction

Mixed valence oxides, of general formula A_1−*x*_M_*x*_FeO_3_, with A = La, and M = Ca and Pb, have attracted significant attention due to their interesting structural and physical properties, such as ferromagnetism with metallic conduction, the order of charge, the Colossal magnetoresistance (CMR)^1–23^ and the magnetocaloric effect (MCE).^4–10^ In this context, a great effort has been made on the iron oxides of formula LaFeO_3_ and their derivatives, both from the structural point of view and their physical properties, which have proved to be of interest, for example in the field of gas sensors. Moreover, this kind of material offers a major advantage, which is the possibility of modifying their physical properties by substitutions based on a good choice of the dopant, of the doping site and of the concentration of the dopant.^11–13^ Indeed, doped LaFeO_3_ is known for its electrocatalytic activities.

In this regard, it is important to note that the substitution of LaFeO_3_ compound generates new structural, magnetic and dielectric properties. For example, the insertion of Ca into the A-site, reported by Andoulsi *et al.*^14^ and by Shi *et al.*,^15^ reduced the electrical resistance and improved the magnetic properties. Also, Benali *et al.*^16^ studied the effect of the introduction of lead cations in the A-site of the compound La_0.8_Ca_0.1_FeO_3_, on the structural, dielectric and gas detection properties. They found that insertion of the Pb ion into the A-site can restrict grain size growth and improve the response to ethanol gas. The best response to ethanol was obtained for a level equal to 0.1 mol of Pb. These properties are further improved with the cobalt cation substitution in the B-site of the compound La_0.8_Ca_0.1_Pb_0.1_FeO_3_;^17^ further more detailed investigations^18,19^ have shown that La_1−*x*_Pb_*x*_FeO_3_ systems are of interest for the study thanks to these important physical properties for many applications and their high functional properties.

For this reason, the substitution of this type of compound has attracted a great deal of interest in recent years and several researchers are concentrating on improving these properties by the process of substitution in one or both A and B-sites.^20–22^

Here, we are looking essentially to enhance the electrical and dielectric properties of these materials by replacing the iron cations with those of magnesium. In fact, this is a continuation of the research conducted by our laboratory team to optimize the appropriate composition for a desired application. Therefore, the aim of this manuscript was to study the effect of the insertion of the 2.5% Mg cation into the B-site of the L_a0.8_Ca_0.1_Pb_0.1_FeO_3_ compound, synthesized by the sol–gel method and annealed at 800 °C, on the structural and dielectric properties.

## 2 Experimental details

### 2.1 Sample preparation

The sol–gel technique^23,24^ is a process for developing solid nano-powder materials from an aqueous solution containing the starting precursors. This process is carried out under so-called soft chemistry conditions (temperatures very close to room temperature). The reaction takes place according to the following chemical equation:0.8La(NO_3_)_3_ + 0.1Ca(NO_3_)_2_ + 0.1Pb(NO_3_)_2_ + 0.975Fe(NO_3_)_2_ + 0.025Mg(NO_3_)_2_ → La_0.8_Ca_0.1_Pb_0.1_Fe_0.975_Mg_0.025_O_3_ + gases

The La_0.8_Ca_0.1_Pb_0.1_Fe_0.975_Mg_0.025_O_3_ compound was prepared by mixing stoichiometric amounts of La(NO_3_)_3_, Ca(NO_3_)_2_, Pb(NO_3_)_2_, Fe(NO_3_)_3_ and Mg(NO_3_)_2_ in distilled water (all reagents are obtained from Sigma-Aldrich with a purity higher than 99.6%). The citric acid was added with maintaining a ratio of *n* (La^3+^, Ca^2+^, Pb^2+^, Fe^3+^, and Mg^2+^) : *n* (citric acid) = 1 : 2. The ethylene glycol was added to further assure the homogeneity of the solution. The obtained mixture was then heated at 70 °C until a viscous brown gel was formed. Heated at 170 °C, the gel was slowly transformed into a fine black powder and heated for 12 hours at 300 °C in order to decompose the organic residuals. Then, the resulting powder was ground and using an uniaxial pressure system (approximately 2 tones per cm^2^), pellets of approximately 8 mm in diameter and 1.8 mm in thickness were made and submitted to heat treatments at different temperatures of 600 °C (12 hours) and 800 °C (12 hours).The phase purity of the prepared compound was checked by X-Ray Diffraction (XRD) analysis (Siemens D5000 X-ray diffractometer, with monochromatic Cu-Kα radiation (*λ* = 1.5406 Å)).

### 2.2 Characterization tools

The X-Ray Diffraction (XRD) patterns of the prepared powder were recorded at room temperature using a Siemens D5000 diffractometer with a copper anticathode over the 10°–100° Bragg angle range. The morphology of the sample was examined using a TESCAN VEGA3 SBH scanning electron microscope (SEM) equipped with an EDS detector BrukerXFlash 410 M for analysing the elemental composition and homogeneity of the prepared sample.

For dielectric measurements, two conducting silver layers were coated on both sides of the prepared pellet. Then, the transport properties of the studied materials were measured using an Agilent 4294A in the frequency range 100 Hz–1 MHz and the temperature range 200 K–400 K using a nitrogen bath cryostat setup. During the measurements, the samples were kept under a helium atmosphere to minimize thermal gradients and the temperature of the samples was controlled using an Oxford Research IT-C4 and measured using a platinum sensor.^25–27^

## 3 Structural characterization

### 3.1 Structural study

The purity and good crystallization of our synthetized compound were systematically verified using a diffractometer driven from a copper anticathode and a back graphite monochromator. X-ray diffractogram recording was performed at room temperature. The angular range varies from 10° to 90° and the recording time is 10 seconds in steps of 0.02°. The used refinement program, FULLPROF,^28^ is derived from the Rietveld method.

**Fig. 1:**
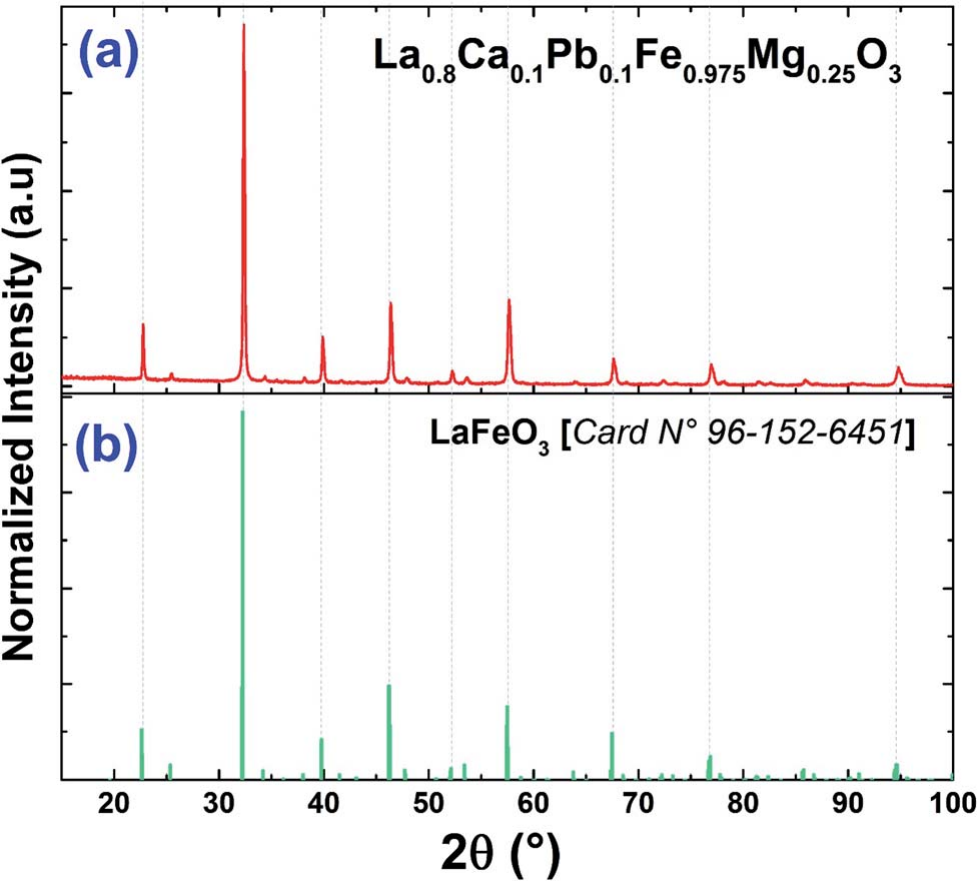
X-ray diffractograms of (a) the La_0.8_Ca_0.1_Pb_0.1_Fe_0.975_Mg_0.025_O_3_ compound and (b) the standard data of LaFeO_3_.^49^

Fig. 1 reveals that the diffractogram obtained from the compound La_0.8_Ca_0.1_Pb_0.1_Fe_0.975_Mg_0.025_O_3_ is almost identical to that of the compound La_0.8_Ca_0.1_Pb_0.1_FeO_3_ studied by A. Benali *et al.*^29^ with a slight modification of the positions of the peaks following the insertion of magnesium.

Recall that Benali *et al.* confirmed the existence of two Ca_2_Fe_2_O_5_ and Fe_3_O_4_ secondary phases in addition to the La_0.8_Ca_0.1_Pb_0.1_FeO_3_ main phase detected using X-pert Highscore phase identification software. These secondary phases are at very low percentages compared to the main phase. We note that the substitution of iron by magnesium in the La_0.8_Ca_0.1_Pb_0.1_Fe_0.975_Mg_0.025_O_3_ compound does not modify the main phase and the secondary phases, which remain the same as those found for the case of the parent compound. As can be seen, the peak intensities of the two secondary phases decrease following the substitution of iron by magnesium.

**Fig. 2:**
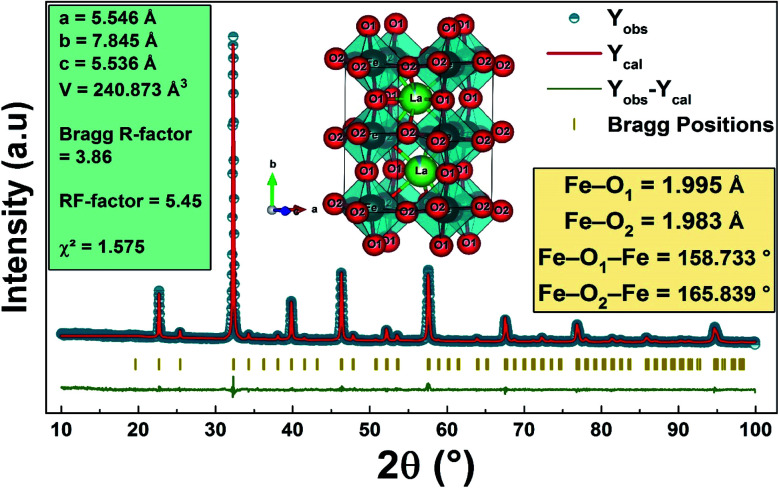
The XRD Rietveld refinement results of the La_0.8_Ca_0.1_Pb_0.1_Fe_0.975_Mg_0.025_O_3_ compound. The inset of the figure presents the generated crystal structure and representation of the FeO_6_ polyhedron of the studied compound.

Fig. 2 shows the refinement diagram of the La_0.8_Ca_0.1_Pb_0.1_Fe_0.975_Mg_0.025_O_3_ compound, where the experimental diffractogram is represented in red, the calculated one in a solid black line, their difference in blue and the positions of the Bragg lines in green. The main phase refinement was performed following the orthorhombic structure with the *Pnma* space group. The main phase refinement results are summarized in Table 1. The substitution of iron by magnesium in the La_0.8_Ca_0.1_Pb_0.1_FeO_3_ compound generates the reduction of the mesh parameters and of its volume.

**Table 1:** Rietveld (RX) refinement results of La_0.8_Ca_0.1_ Pb_0.1_Fe_1−*x*_ Mg_*x*_O_3_ compounds (*x* = 0.00 and *x* = 0.025)

*X*	0.00	0.025
Space group	*Pbnm*	*Pbnm*
*a* (Å)	5.539 (1)	5.546 (0)
*b* (Å)	7.831 (0)	7.845 (3)
*c* (Å)	5.543 (4)	5.536 (0)
*V* (Å^3^)	60.120 (6)	60.218 (2)

### 3.2 Calculation of grain size

The grain size was calculated by Scherrer's formula^30^ based on the X-ray diffraction data of the most intense peak:1
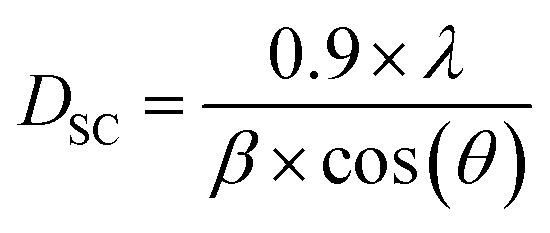
with *λ* = 1.5406 Å, *θ*: the most intense peak diffraction angle, and *β*: the width at mid-height (rad).

The average crystallite size obtained from Scherrer's formula was found to be equal to DSC = 40.87 nm while the grain size of the parent compound La_0.8_Ca_0.1_Pb_0.1_FeO_3_ is DSC = 37.42 nm. It is clear then that the insertion of magnesium with an atomic radius greater than that of iron, even at a low concentration, increases the grain size.

### 3.3 Calculation of porosity

In order to evaluate the porosity *p* of our compounds, we started by calculating the density *d*_x_ of the compounds from the following relation:^31^2
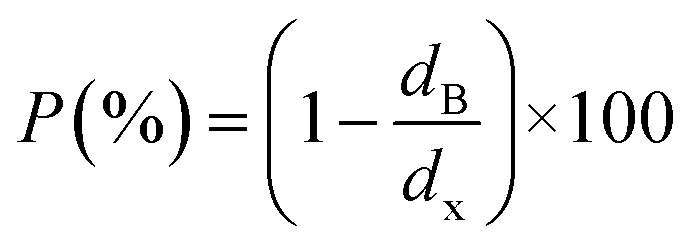
3
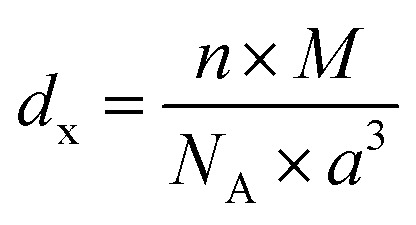
4
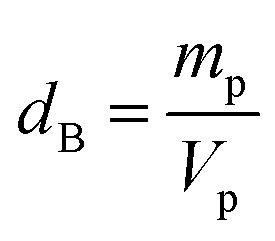
where *M* is the molecular mass, *N*_A_ is the Avogadro number, *a* is the lattice parameter, *m* is the mass of the powder used for characterization and *V*_p_ is the total volume it occupies. The values are summarized in Table 2. The porosity for the studied compound is around 61.59%.

**Table 2:** The values of the apparent density (*d*), the X-ray density (*d*_x_) and the porosity (*p*) of the La_0.8_Ca_0.1_Pb_0.1_Fe_0.975_Mg_0.025_O_3_ compound

*X*	*d*_x_ (g cm^−3^)	*d* (g cm^−3^)	Porosity *p* (%)
0.00	2.333	0.838	64.08
0.025	2.181	0.838	61.59

### 3.4 Morphological study

After preparation, a morphological study with a scanning electron microscope of the TESCAN VEGA3 SBH type is carried out on the La_0.8_Ca_0.1_Pb_0.1_Fe_0.975_Mg_0.025_O_3_ compound, in order to have an overview on the particle size and the morphology of the compound surface.

**Fig. 3:**
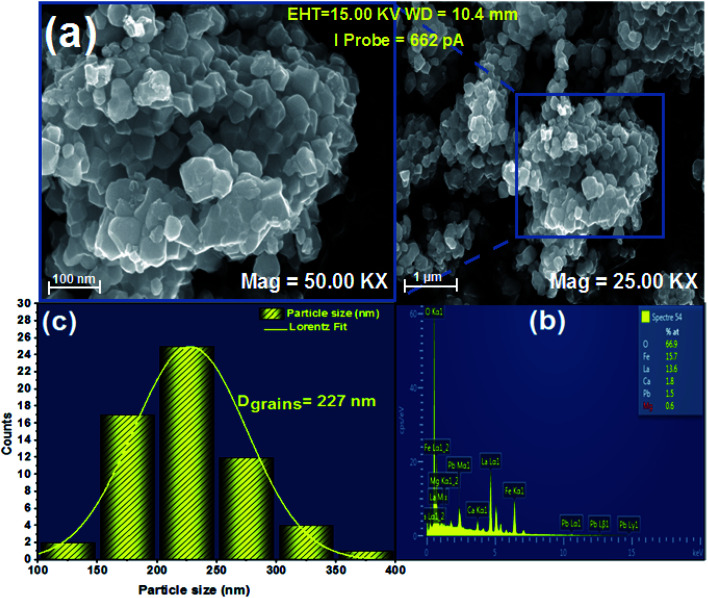
(a) SEM image, (b) EDX analysis of the compound La_0.8_Ca_0.1_Pb_0.1_Fe_0.975_Mg_0.025_O_3_ and (c) size distribution histogram.

Fig. 3(a) shows that the grains are contiguous and present different polygonal and cubic shapes. The average particle size was calculated from these images using “Image J” software which allowed us to plot a histogram of the average size as a function of the number of grains considered as shown in Fig. 3(c).

The average particle size determined from the SEM image is larger than that calculated using the Scherrer formula (DSC). This difference is probably due to the fact that each grain observed by the SEM is made up of a set of small-crystallized grains.

The elemental compositions of the synthesized sample were carefully analysed by energy dispersive spectroscopy (EDS), as shown in Fig. 3(b). This spectrum allowed the detection of the presence of all chemical elements for our compound. This shows the success of Mg-substitution in the La_0.8_Ca_0.1_Pb_0.1_Fe_0.975_Mg_0.025_O_3_ compound.

## 4 Dielectric study

### 4.1 *Z*′′in terms of *Z*′′

**Fig. 4:**
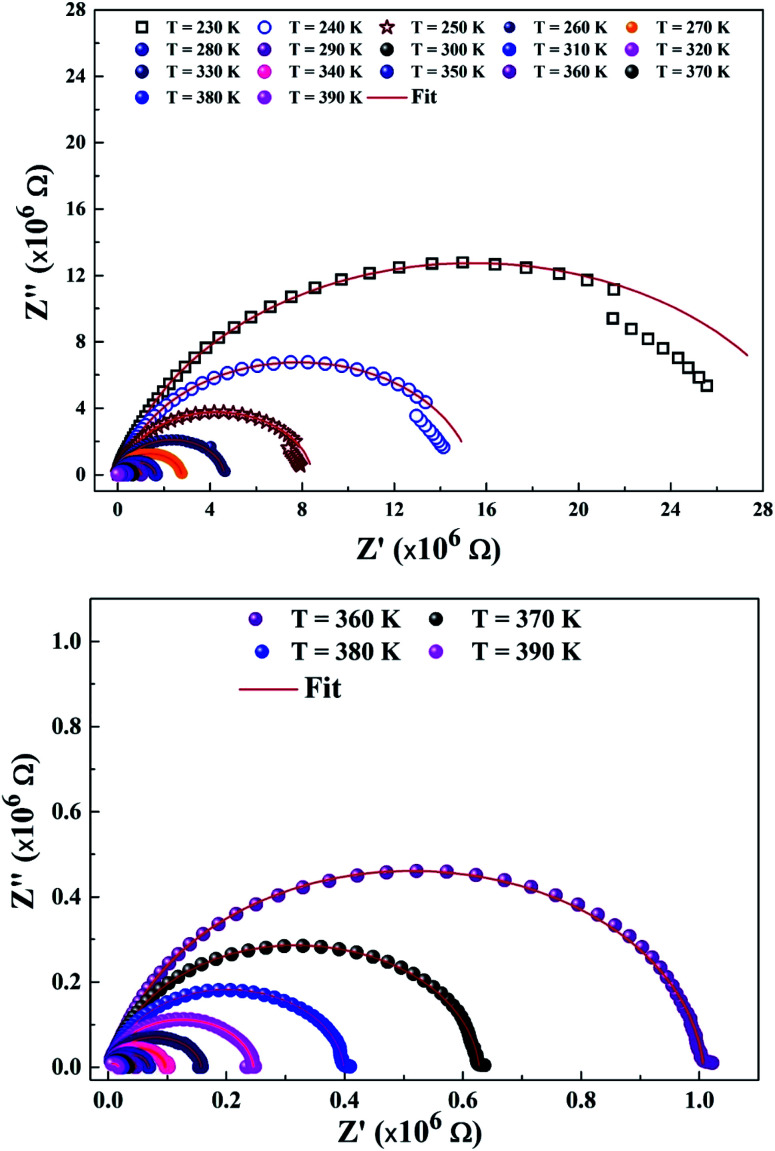
Nyquist diagram of the La_0.8_Ca_0.1_Pb_0.1_Fe_0.975_Mg_0.025_O_3_ compound.

**Fig. 5:**
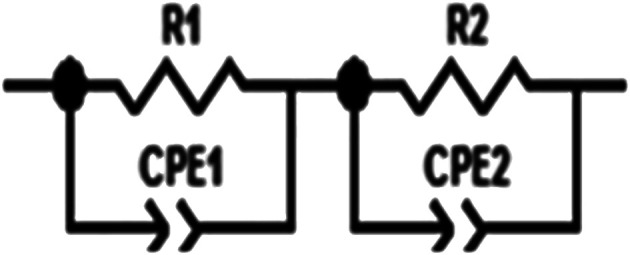
Equivalent circuit of the La_0.8_Ca_0.1_Pb_0.1_Fe_0.975_ Mg_0.025_O_3_ compound.

To explain the electrical behavior of a material, Bauerle proposed simple electrical circuits made up of resistors and capacitors.^32^ The equivalent circuit, therefore, makes it possible to establish a correlation between the electrochemical parameters and the characteristic elements of the impedance. Indeed, in order to take into account the physical phenomena responsible for conduction and relaxation phenomena, we analysed the real and imaginary parts of the impedance data using Z-view software.^33^ We show in Fig. 4 the variation of the imaginary part of impedance *Z*′′ as a function of the real part of impedance *Z*′ for different temperatures. In accordance with literature results, the best fit of the impedance Nyquist plots was found when using two combinations of two electrical cells; each one contains a resistance (*R*) connected in parallel with a capacitance (CPE) as described in Fig. 5. Each electric cell describes the electrical properties and the relaxation process of one contribution in the La_0.8_Ca_0.1_Pb_0.1_Fe_0.975_Mg_0.025_O_3_ compound. Note that the two contributions are the grain and grain boundary and that the error of all calculated parameters (Table 3) was less than 4%.

**Table 3:** Parameters of the equivalent circuit

*T* (K)	*R*_jg_ (×10^5^ Ω)	CPE _jg_ (×10^−11^F)	*α*	*R*_g_ (×10^3^ Ω)	*C*_g_ (×10^−10^ F)	*α*
240	150	2.3	0.92	353.870	1.2	0.88
250	82.8	2.27	0.93	167.900	1.07	0.889
260	45.1	2.05	0.96	66.125	1.18	0.875
270	26.8	2.05	0.95	114.700	1.1	0.877
280	15.7	1.86	0.96	48.630	1.6	0.849
290	9.822	2.03	0.957	24.528	1.63	0.860
300	6.140	2.15	0.954	13.492	1.15	0.888
310	3.889	2.2	0.9546	9.492	2.06	0.853
320	2.40340	2.44	0.95	5.507	3.38	0.820
330	1.55040	2.82	0.938	2.462	0.253	0.983
340	0.91766	2.07	0.966	4.783	70.9	0.657
350	0.56745	1.77	0.98	11.926	51.6	0.674
360	0.36700	1.65	0.996	9.720	53.3	0.674
370	0.31720	2.72	0.947	0.506	0.267	1.358
380	0.23444	2.13	0.965	0.706	0.152	0.5
390	0.17185	2.437	1.7185	0.510	4.4	0.959

In the low frequency region, the semicircle with high diameter corresponds to the grain boundary contribution (*R*_gb_) whereas at higher frequencies the electrical response is due to the grain contribution (*R*_g_) as it previously confirmed.^34,35^

From the results regrouped in Table 3, one can see that the grain boundary resistance (*R*_gb_) values are much higher than those of the grains (*R*_g_) and that both resistance values decreased by increasing the temperature. This confirms well the p-type semiconductor nature of the La_0.8_Ca_0.1_Pb_0.1_Fe_0.975_Mg_0.025_O_3_ compound.

**Fig. 6:**
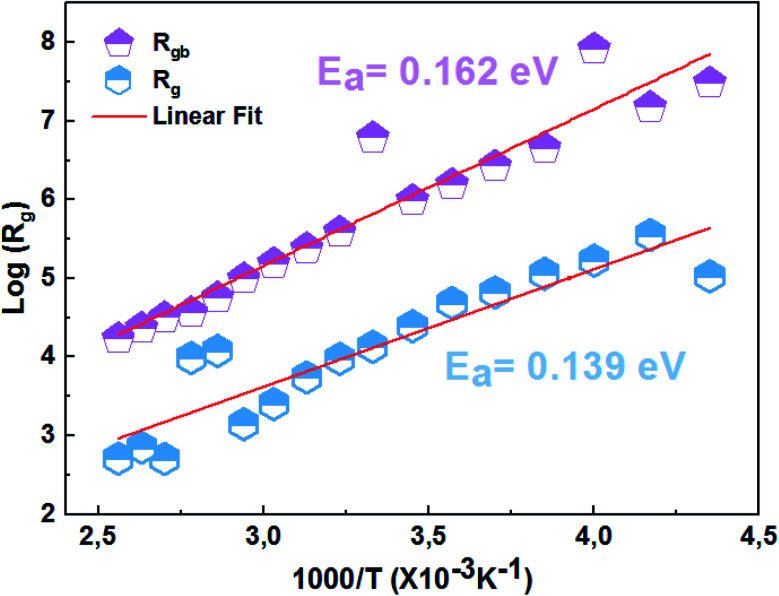
Log(*R*) variation as a function of 1000/*T* of grain contributions and grain boundaries of the La_0.8_Ca_0.1_Pb_0.1_Fe_0.975_Mg_0.025_O_3_ compound.

Furthermore, we present in Fig. 6 the logarithmic variation of both *R*_gb_ and *R*_g_ as a function of 1000/*T*. For both contributions, we found that the resistance linearly follows the increase in temperature, indicating an Arrhenius power law as can be described as follows:^36^5
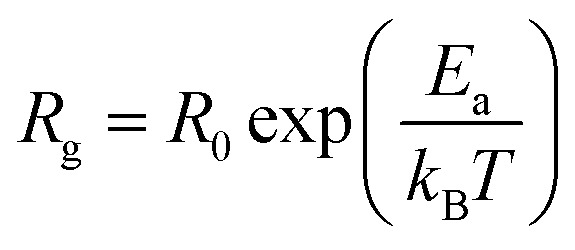


It can be seen that the calculated activation energy value related to the grain boundary contribution is greater than that of the grain contribution.

### 4.2 Variation of the real part of impedance *Z*′

**Fig. 7:**
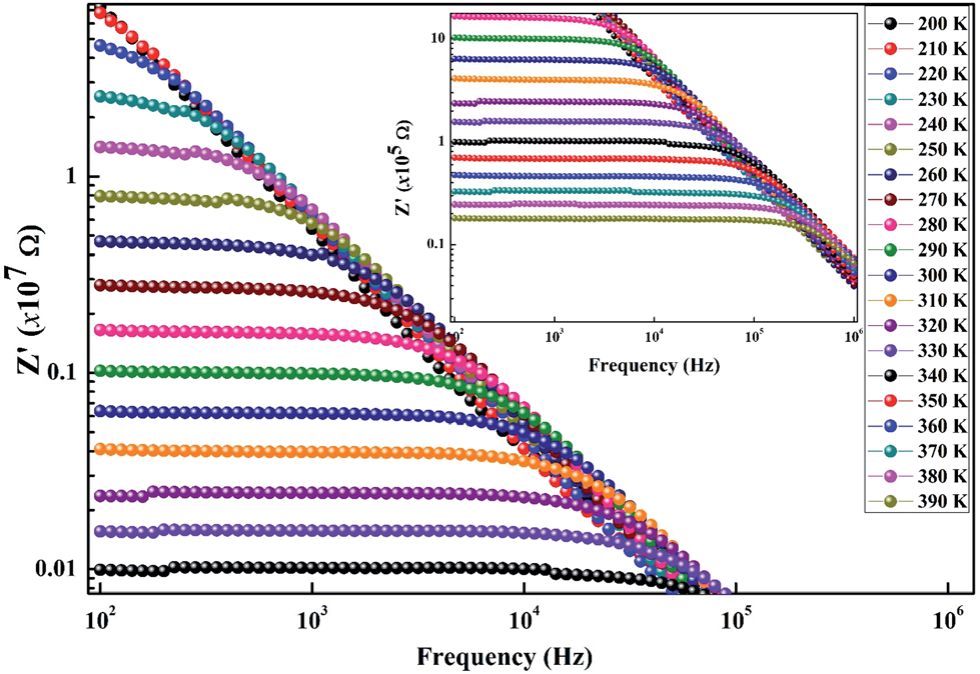
Variation of the real impedance part (*Z*′) as a function of the frequency at different temperatures of the La_0.8_Ca_0.1_Pb_0.1_Fe_0.975_Mg_0.025_O_3_ compound.

Fig. 7 presents the variation of the real part of impedance (*Z*′) as a function of the frequency at different temperatures of the La_0.8_Ca_0.1_Pb_0.1_Fe_0.975_Mg_0.025_O_3_ compound. As can be seen in Fig. 7, the real part of the impedance decreases with increasing frequency and temperature, indicating the increase in conductivity *σ*_ac_.^37^ At higher frequencies, the coincidence of impedance (*Z*′) for all temperatures indicates a possible release of space charges.^38^

Considering the equivalent circuit adopted we can write the characteristic equation of the variation of the real part of impedance *Z*′.6
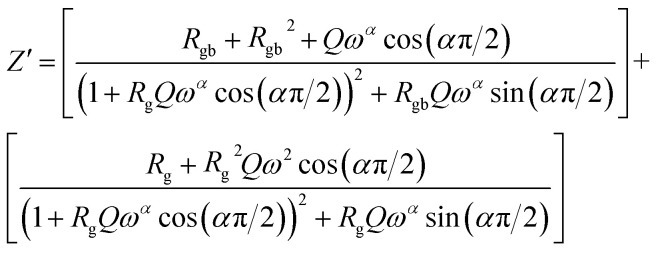


### 4.3 Variation of the imaginary part of impedance *Z*′′

**Fig. 8:**
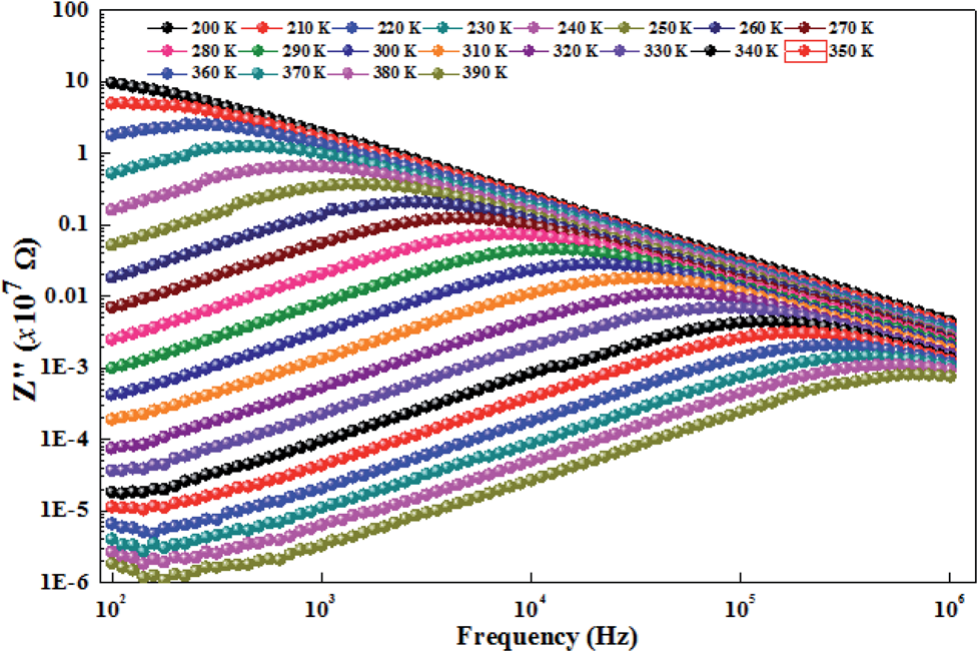
Variation of the imaginary impedance part (*Z*′′) as a function of the frequency at different temperatures of the La_0.8_Ca_0.1_Pb_0.1_Fe_0.975_Mg_0.025_O_3_ compound.

To better understand the relaxation phenomenon in the La_0.8_Ca_0.1_Pb_0.1_Fe_0.975_Mg_0.025_O_3_ compound, we have presented in Fig. 8 the variation of the imaginary part of impedance (*Z*′′) as a function of the frequency at different temperatures. By examining these curves, we observe the appearance of a relaxation peak (*Z*′′_max_), which moves towards high frequencies when the temperature increases. According to the literature,^39–42^ the displacement of the relaxation peak confirms the existence of a Debye relaxation phenomenon within the material. This process is probably due to the presence of electrons and/or stationary space charges at low temperatures.^43^

**Fig. 9:**
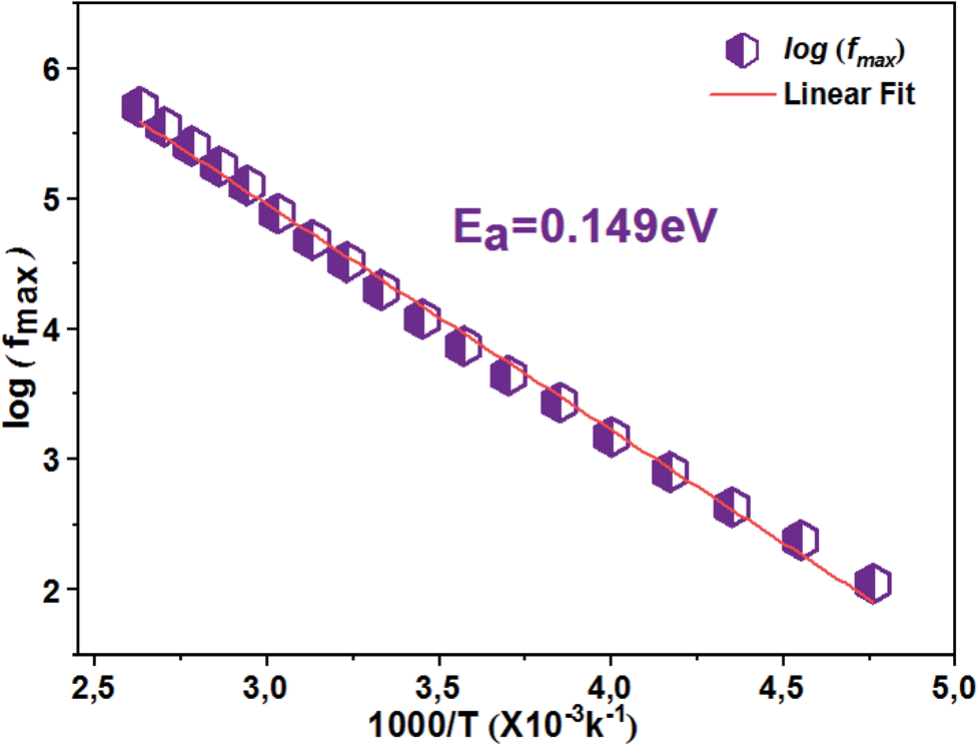
Thermal variation of log(*f*_max_) of the La_0.8_Ca_0.1_Pb_0.1_Fe_0.975_Mg_0.025_O_3_ compound.

The thermal variation of log(*f*_max_) (Fig. 9) is linear which follows Arrhenius' law:7
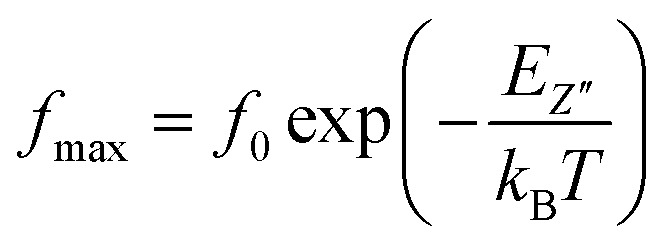


From this variation, we have deduced that the activation energy is around *E*_a_ = 0.149 eV.

### 4.4 Complex modulus analysis

The complex modulus can be written as a real component *M*′ and an imaginary component:

8 M* = 1/ε* = *jωC*_0_Z* = *M*′ + *jM*′′

9 *M*′′ = *ωC*_0_*Z*′, *C*_0_ = *ε*_0_*S*/*e*

where *ε*_0_ is the electrical permittivity of vacuum, *S* is the surface of the sample (0.503 cm^2^), *e* is the thickness of the sample (*e* = 1.25 mm) and *ω* = 2π*f*: pulsation.

**Fig. 10:**
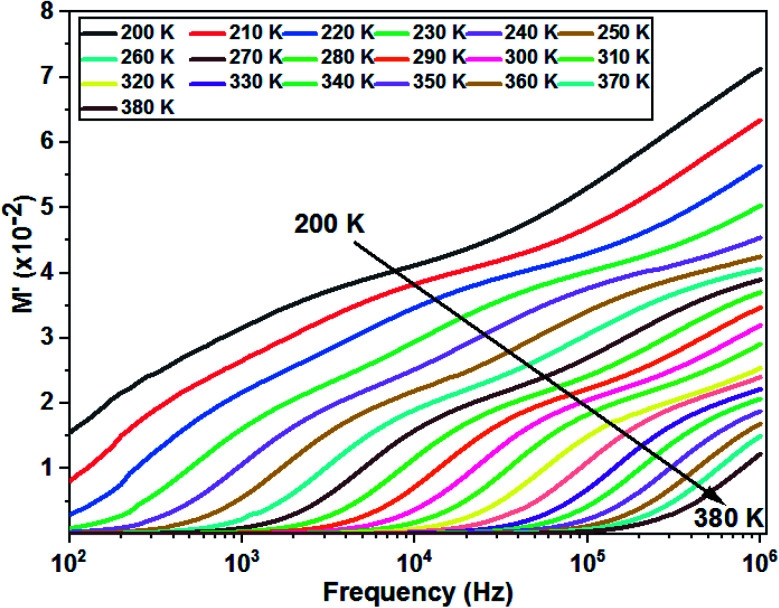
Variation of the real part of modulus as a function of the frequency at different temperatures of the La_0.8_Ca_0.1_Pb_0.1_Fe_0.975_Mg_0.025_O_3_ compound.

Fig. 10 shows the variation of *M*′ as a function of frequency for different temperatures. The curves clearly show a very low *M*′ value in the low frequency region and an increase in the value of *M*′ in the high frequency region. In addition, the value of *M*′ decreases with increasing temperature and the curves are well dispersed in which the region of dispersion shifts towards higher frequencies with an increase in temperature. This behavior supports the long-distance mobility of charge carriers. The dispersive nature of the modulus implies a well-defined relaxation mechanism over several decades of frequency for all temperatures.

**Fig. 11:**
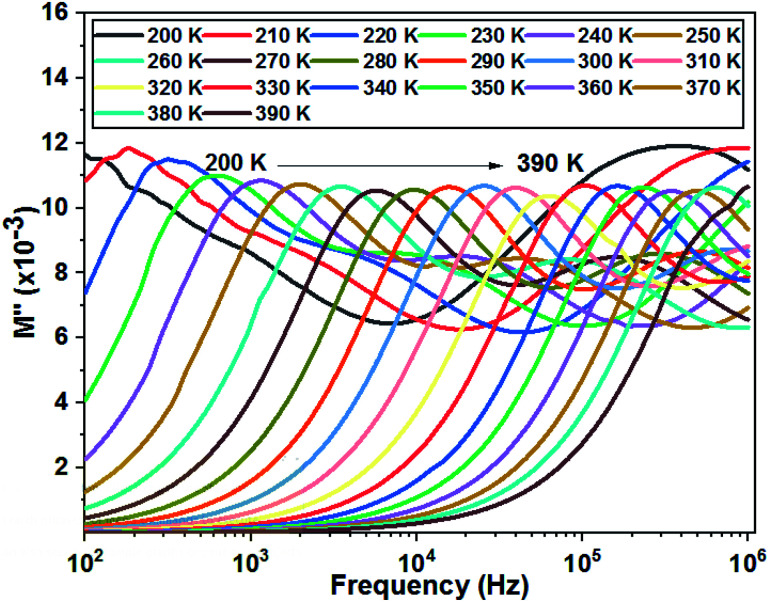
Variation of the imaginary part of modulus as a function of the frequency at different temperatures of the La_0.8_Ca_0.1_Pb_0.1_Fe_0.975_Mg_0.025_O_3_ compound.

Fig. 11 shows the variation of the imaginary part of the complex modulus (*M*′′) with frequency at different temperatures. The *M*′′(*f*) curves are characterized by well resolved peaks in the pattern occurring at a single frequency for each temperature. Those at low frequencies are associated with grain boundary effects and the peaks observed towards high frequencies are correlated with grain contribution.

**Fig. 12:**
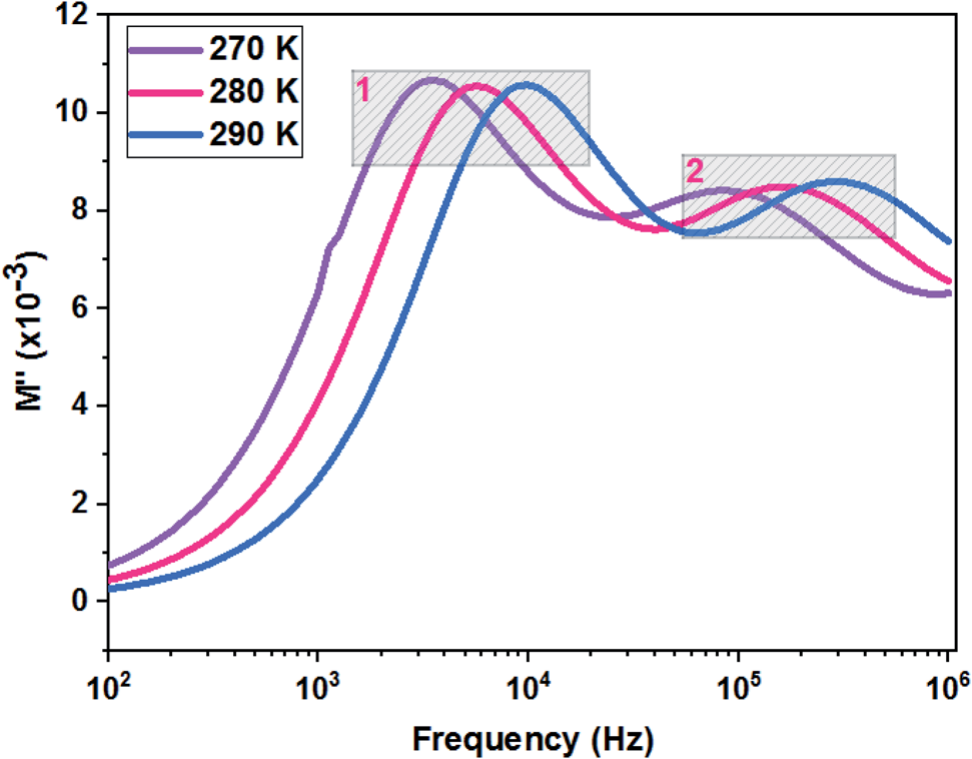
Relaxation peaks: **1** are associated with grain boundary effects and **2** are correlated with grain contribution of the La_0.8_Ca_0.1_Pb_0.1_Fe_0.975_Mg_0.025_O_3_ compound.

The logarithmic variations of the frequencies corresponding to the peaks as a function of the inverse of the temperature are given in Fig. 12. These variations represent straight lines following the Arrhenius law, whose slopes will be used to calculate the activation energy:10
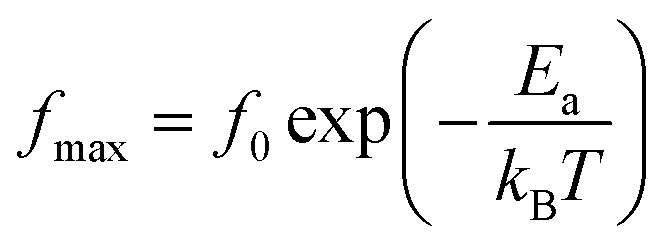


**Fig. 13:**
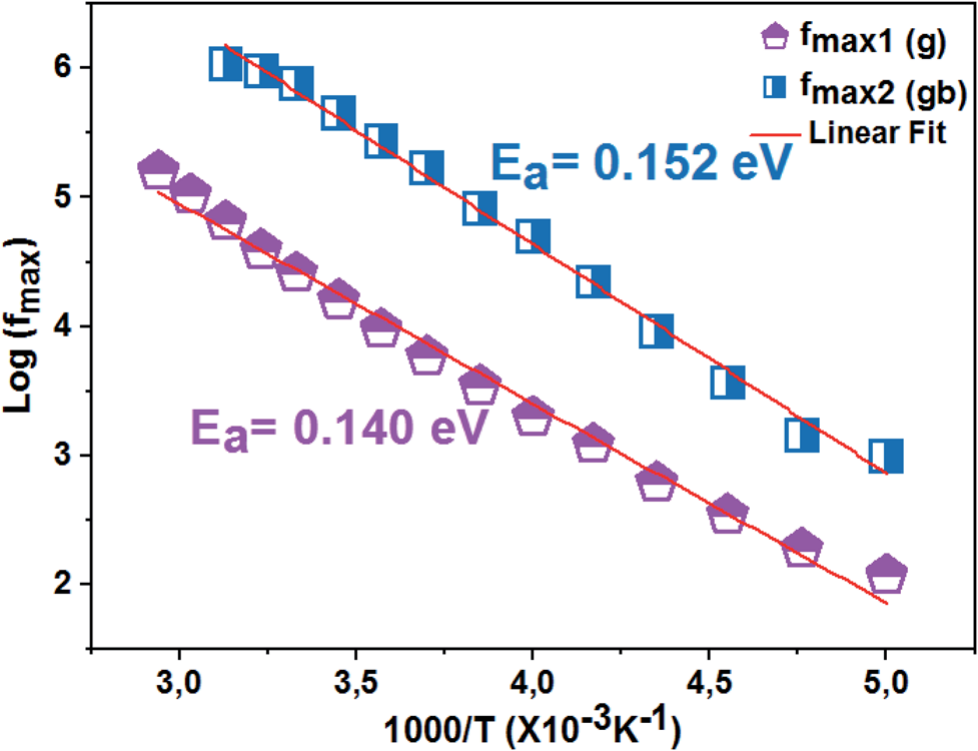
Variation of log(*f*) as a function of 1000/*T* of the La_0.8_Ca_0.1_Pb_0.1_Fe_0.975_Mg_0.025_O_3_ compound.

We deduce from the slopes of the lines of log(*f*) as a function of temperature the activation energies of grains (*E*_g_ = 0.140 eV) and grain boundaries (*E*_gb_ = 0.152 eV). These two values are very close to those determined previously from the resistances of two contributions and from *Z*′′ curves. This proves the good fit of the Nyquist and that the equivalent circuit is the most adequate (Fig. 13).

### 4.5 Loss factor variation as a function of frequency

**Fig. 14:**
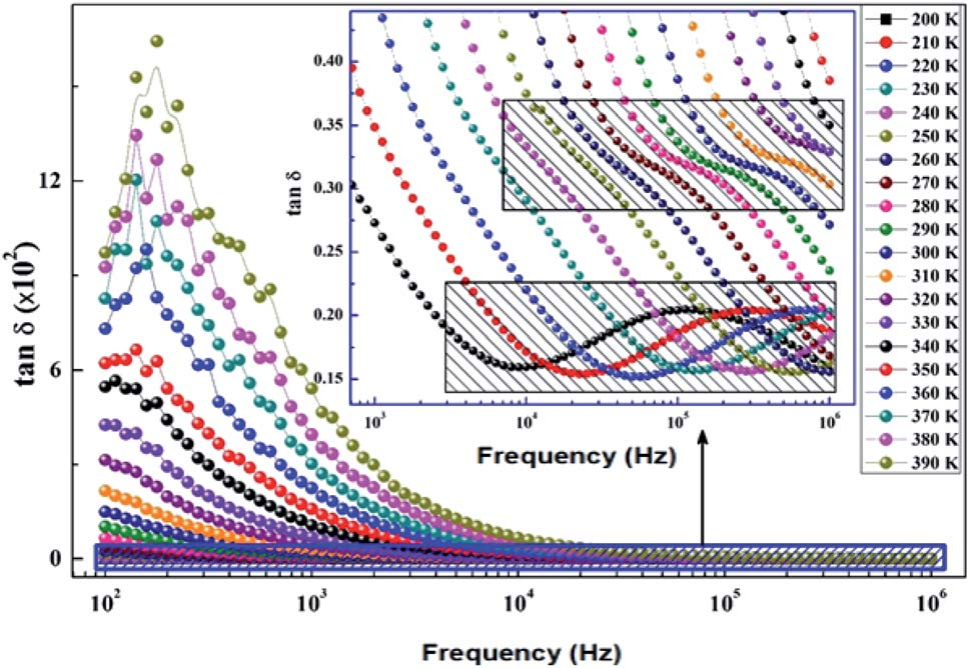
Variation of dielectric losses tg(*δ*) as a function of frequency at different temperatures of the La_0.8_Ca_0.1_Pb_0.1_Fe_0.975_Mg_0.025_O_3_ compound.

The dissipation factor tg(*δ*) is a complementary element for the study of the permittivity in order to determine the presence of the relaxation phenomenon in the material. Fig. 14 presents the variation of the loss factor tg(*δ*) as a function of the frequency at different temperatures. These curves clearly show the increase in dielectric losses in the low frequency region and the decrease in the latter in the high frequency region.

**Fig. 15:**
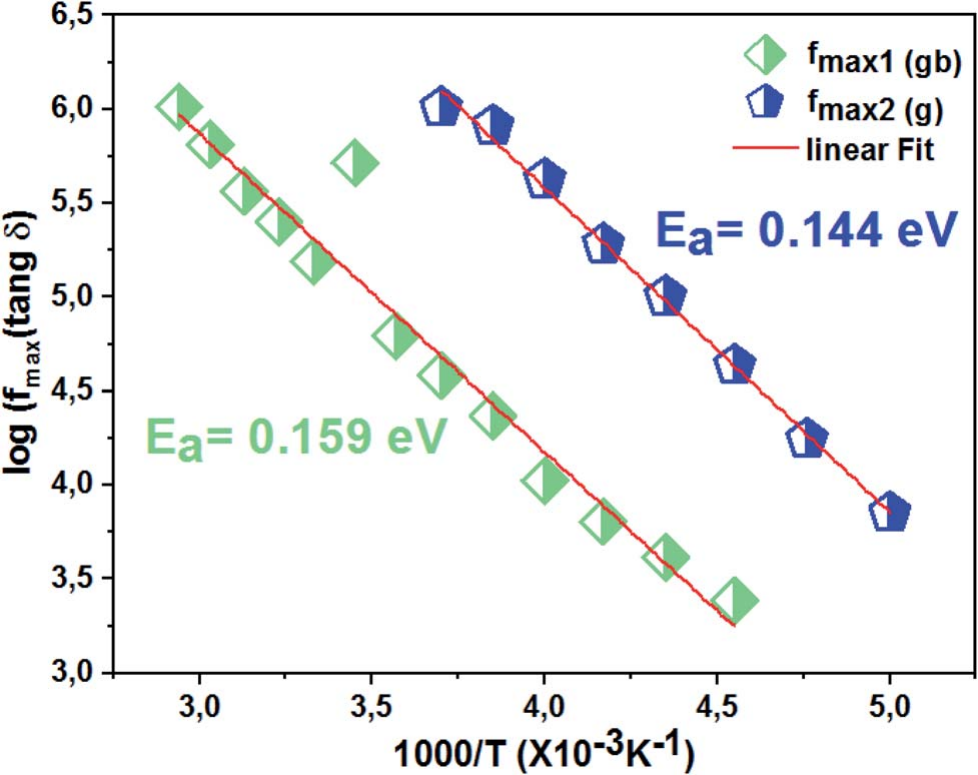
Thermal variation of log(*f*) of the La_0.8_Ca_0.1_Pb_0.1_Fe_0.975_Mg_0.025_O_3_ compound.

To determine the activation energy (*E*_a_) we plot the curves of log(*f*) = *f*(1/*T*) (Fig. 15). The *E*_a_ values, calculated from the slopes of these curves, are found to be equal to *E*_g_ = 0.144 eV for the grains and *E*_gb_ = 0.159 eV for the grain boundaries.

### 4.6 Dependence of conductivity on frequency

**Fig. 16:**
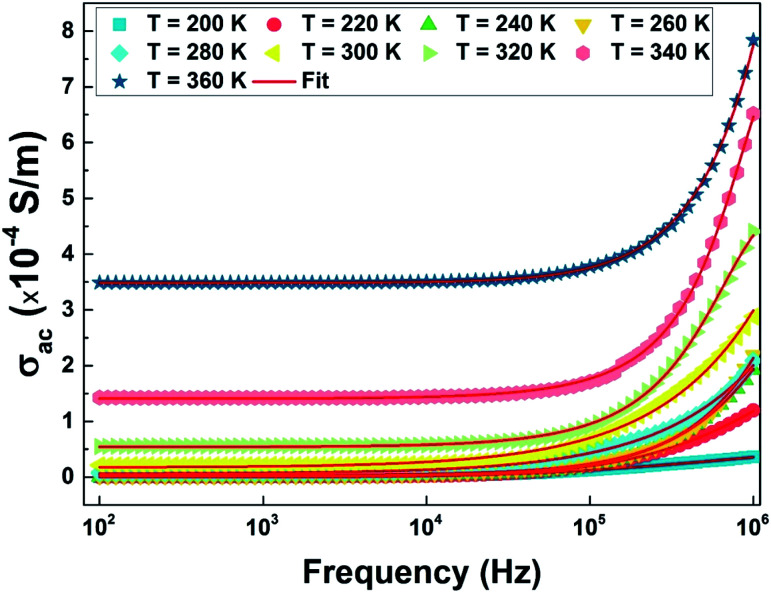
The frequency and temperature dependence of the ac-conductivity (*σ*_ac_) and their respective adjustment results according to Jonscher's power law (red line) of the La_0.8_Ca_0.1_Pb_0.1_Fe_0.975_Mg_0.025_O_3_ compound.

The measurement of the conductivity *σ*ac (Fig. 16) was carried out using impedance spectroscopy in the frequency range of 40 Hz–1 Mz and a temperature range of 100 to 400 K.

We notice that at low frequency, the conductivity is independent of the frequency and has a wide plateau corresponding to a uniform conductance dependent on the temperature. In this case, only the diffusive aspect of ionic transport is considered and the conductivity *σ*_ac_ is equal to the static conductivity (*σ*_ac_ = *σ*_dc_). On the other hand, we note that the value of *σ*_ac_ increases with increasing temperature which is a characteristic of a thermally activated conduction process. However, at high frequencies the value of conductivity increases with frequency according to a power law *Aω*^*s*^. This regime represents the displacement current, and the conductivity is equal to the dynamic conductivity *σ*_dc_.

Consequently, the conductivity *σ*_ac_ of our compound is described according to the approach of Jonscher's law^44^ by the sum of two terms and is then written as

11 *σ*_ac_ = *σ*_dc_ + *Aω*^*s*^

where *ω* is the frequency, and *σ*_dc_ is the low frequency conductivity which depends on the temperature and *s* (0 ≤ *s* < 1).

The exponent *s* is related to the variation of the polarizability induced in the material. This dimensionless parameter is generally used to characterize the electrical conduction mechanism throughout the material. In the high frequency range, the conductivity *σ*_ac_ obeys the type of law:

12 *σ*_ac_(*ω*) = *Aω*^*s*^

This type of variation is characteristic of the jump conduction process. The increase in conductance with temperature indicates thermal activation of the conduction process in this material.

We performed an adjustment of the curves as shown in Fig. 16 using eqn (12) with ORIGIN8.0 software. The refinement results are shown in Table 4.

**Table 4:** Results of refinements of the conductivity of the La_0.8_Ca_0.1_Pb_0.1_Fe_0.975_Mg_0.025_O_3_ compound

*T* (K)	*σ*_dc_ (S m^−1^)	*A*	*S*
200	8.050 × 10^−7^	2.996 × 10^−10^	0.454
220	7.123 × 10^−7^	3.639 × 10^−10^	0.497
240	6.562 × 10^−6^	3.933 × 10^−10^	0.527
260	1.654 × 10^−6^	6.133 × 10^−10^	0.571
280	8.731 × 10^−5^	9.553 × 10^−10^	0.612
300	3.726 × 10^−5^	1.112 × 10^−9^	0.648
320	1.098 × 10^−5^	6.023 × 10^−9^	0.699
340	7.906 × 10^−4^	7.906 × 10^−9^	0.761
360	2.487 × 10^−4^	2.487 × 10^−9^	0.791

By examining the values of the parameter “*s*” we notice that it increases with increasing temperature.^45^ Hence, the small tunnel polaron jump model (NSPT) is the most suitable model.

According to this model, the variation of this parameter as a function of the temperature can be written as follows:^46–48^13
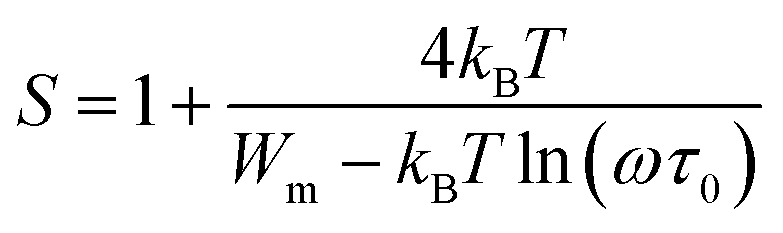
where *W*_m_ is the binding energy of the charge carrier in its localized sites and *k*_B_ is the Boltzmann constant.

For large values of *W*_m_/*k*_B_*T*, this equation becomes14
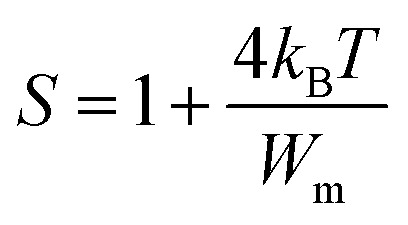


**Fig. 17:**
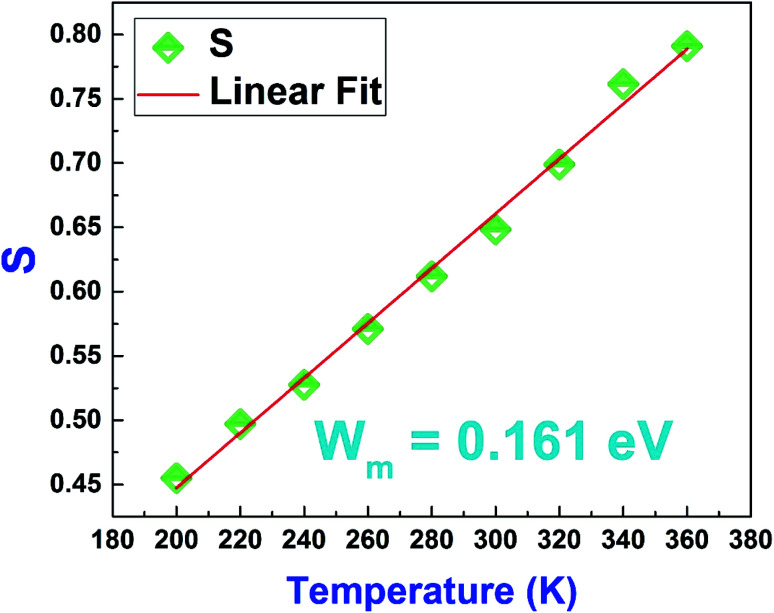
Variation of the Jonscher's power law exponent “*S*” as a function of temperature of the La_0.8_Ca_0.1_Pb_0.1_Fe_0.975_Mg_0.025_O_3_ compound.

In order to determine *W*_m_, we have plotted in Fig. 17 the variation of *S* as a function of temperature.

Based on equation eqn (14), the values of *W*_m_ (polaron energy) are determined using the slope of the curves *S*(*T*) and the obtained value was found to be around 0.161 eV.

## 5 Conclusion

This work focused on the development and study of the physical properties of the La_0.8_Ca_0.1_Pb_0.1_Fe_0.975_ Mg_0.025_O_3_ compound, which has been characterized by various experimental techniques such as X-ray diffraction, SEM and complex impedance spectroscopy. The XRD study showed that this compound crystallized in the orthorhombic structure with the *Pnma* space group. It is found that the grain sizes, determined from the SEM images, are much greater than those calculated by the Scherrer formula (DSC). This difference was related to the fact that each grain observed by the SEM is made up of a set of small crystallized grains. The study of the variation of the real part *Z*′ and imaginary *Z*′′ of the complex impedance of the La_0.8_Ca_0.1_Pb_0.1_Fe_0.975_ compound as a function of the frequency allowed us to define the equivalent circuit. The circuit chosen is made up of two cells connected in series. The first circuit is formed by a parallel combination of a resistance *R*_1_ and a fractal capacitance CPE_1_ related to the contribution of the grains. The second one consists of a resistor *R*_2_ and a fractal capacitor CPE_2_ mounted in parallel, which corresponds to the contribution of the grain boundaries. From the impedance spectroscopy measurements, we determined the different dielectric parameters to know the conductivity *σ*_ac_, the complex impedance *Z*, the complex modulus *M* and dielectric losses. The electrical properties of our materials have been found to be highly dependent on temperature and frequency.

## Conflicts of interest

There are no conflicts to declare.

## Supplementary Material
